# Philosophy of the Spike: Rate-Based vs. Spike-Based Theories of the Brain

**DOI:** 10.3389/fnsys.2015.00151

**Published:** 2015-11-10

**Authors:** Romain Brette

**Affiliations:** ^1^UMR_S 968, Institut de la Vision, Sorbonne Universités, UPMC University, Paris 06Paris, France; ^2^INSERM, U968Paris, France; ^3^CNRS, UMR_7210Paris, France

**Keywords:** action potentials, firing rate, information, neural coding, neural computation, neural variability, spike timing

## Abstract

Does the brain use a firing rate code or a spike timing code? Considering this controversial question from an epistemological perspective, I argue that progress has been hampered by its problematic phrasing. It takes the perspective of an external observer looking at whether those two observables vary with stimuli, and thereby misses the relevant question: which one has a causal role in neural activity? When rephrased in a more meaningful way, the rate-based view appears as an *ad hoc* methodological postulate, one that is practical but with virtually no empirical or theoretical support.

## Introduction

Neurons of the central nervous system interact primarily with action potentials or “spikes”, which are stereotyped electrical impulses. In an early electrophysiological experiment in sensory nerve fibers of frog muscles, Adrian and Zotterman (1926) demonstrated that stimulation strength modulated the frequency of spikes produced by the fibers. According to the classical view of neural computation, the atom of information and computation is this “firing rate”, and not the individual spikes. In particular, the precise timing of spikes has little relevance in this view. In contrast, a number of scientists have argued that neural computation critically relies on the temporal coordination of spikes. There is a large diversity of such theories, some based on synchrony, for example synfire chains (Abeles, 1991), polychronization (Izhikevich, 2006), binding by synchrony (Singer, 1999; von der Malsburg, 1999) and synchrony invariants (Brette, 2012), others based on asynchronous firing, for example rank order coding (Thorpe et al., 2001) and predictive spike coding (Deneve, 2008). These two lines of neural theory are broadly categorized as “rate-based” and “spike-based”.

Do individual spikes matter or can neural computation be essentially described in terms of rates, with spikes physically instantiating this description? This contentious question has generated considerable debate in neuroscience, and is still unsettled (Softky and Koch, 1993; Castelo-Branco et al., 1998; Shadlen and Newsome, 1998; Singer, 1999; von der Malsburg, 1999; deCharms and Zador, 2000; Thorpe et al., 2001; Banerjee et al., 2007; London et al., 2010; Randy, 2011). A number of studies have addressed this problem by examining the information (in the sense of Shannon) in spike timing and in firing rate (Petersen et al., 2002; Foffani et al., 2009; Quian Quiroga and Panzeri, 2009); others have looked at empirical evidence supporting specific theories (Engel et al., 2001; Fabre-Thorpe et al., 2001; Ikegaya et al., 2004).

This text is entitled “Philosophy of the spike” because of its epistemological character: its primary goal is to better define the question and understand the nature of the arguments. To this end, I will critically examine three common assertions in this debate:

1. Both rate and spike timing are important for coding, so the truth is in between.
2. Neural responses are variable, therefore neural codes can only be based on rates.
3. The difference between rate-based and spike-based theories is a question of timescale.

It will emerge from this analysis that much of the confusion in this debate comes from casting the question exclusively in terms of coding, that is, in terms of the relationship between stimuli and particular observables (spike trains or rates). However, as I will argue, the question of interest is not whether these observables vary with stimuli, but rather whether they have a causal role in the activity of the nervous system. In other words, can a functional model of the nervous system be based on rates, or are spikes indispensable? Since rates are defined as averages over spikes, this question boils down to whether a realistic spike-based model can be approximated by a rate-based model. I will point out that the possibility of such an approximation requires very stringent conditions, of which there is little or negative evidence.

## Assertion #1: Both Rate and Spike Timing are Important for Coding, so the Truth is in Between

This assertion relies on two implicit assumptions: (1) that rate and spike timing are two concepts of the same nature and (2) that it is possible to conceive an intermediate concept that combines them. Its logic also relies on what is known in philosophy as the “golden mean fallacy”, in reference to Aristotle: between two extreme positions, an intermediate position is more likely to be true. It is a fallacy because the conclusion does not follow from the specific nature of the two positions, but from their being arbitrarily presented as two extremes on a graded scale of value. For example, some people believe that there is a God, others think that there is no God. Does it follow that there is half a God?

The first point I want to establish is that rates and spikes belong to two different conceptual categories, and so there is no middle ground between rate-based and spike-based views. Second, I will show that the confusion in this assertion stems mainly from the term “coding”.

### The Concept of Spike and the Concept of Rate

Neurons mainly communicate with each other using trains of electrical impulses or spikes. Some experimental evidence suggests that spikes are not as stereotyped as we used to think (Debanne et al., 2013). But in any case, they can be characterized as discrete events, with a relatively well-defined time, which we can identify to the time of release of synaptic vesicles at the axonal terminal. Thus, with some qualifications (see “Conclusion” Section), a single spike is associated with physically measurable quantities, and it forms the basis of communication between neurons on a fast timescale. To a large extent (but this is not a key point), it can be characterized by its timing.

On the other hand, in rate-based models of neural activity, the firing rate is an abstract concept that is defined in a limit that involves an infinite number of spikes (of course, experimental measures of firing rate necessarily involve finite numbers of spikes). For example, it can be defined for a single neuron as a temporal average: the inverse of the mean inter-spike interval. There are other definitions, but in all cases, rate is an average quantity defined from the timing of spikes. Thus these are two different concepts: spike timing is what defines spike trains, whereas rate is an abstract mathematical construction on spike trains. Therefore the rate vs. timing debate is not about which one is the relevant quantity, but about whether rate is a sufficiently good description of neural activity or not. Spike-based theories do not necessarily claim that rate does not matter, they refute the notion that rate is the essential quantity that matters.

**Figure 1:**
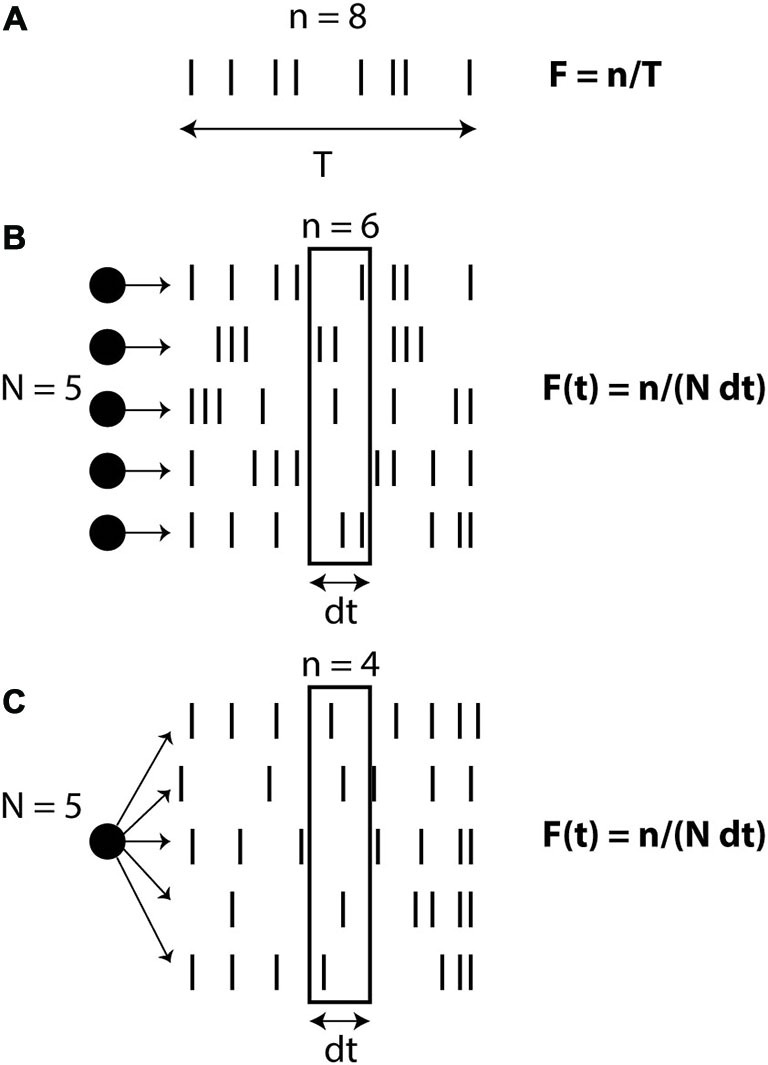
**Definitions of firing rate. (A)** Rate as a temporal average (number of spikes n divided by observation time T). **(B)** Rate as a spatial average over N neurons, on a short time window dt. **(C)** Rate as a probability of firing, corresponding to an average over N trials for the same neuron.

There are different ways of defining the firing rate (Figure 1): over time (number of spikes divided by the duration, in the limit of infinite duration), over neurons (average number of spikes in a population of neurons, in the limit of an infinite number of neurons) or over trials (average number of spikes over an infinite number of trials). In the third definition (which might be the prevailing view), the rate is seen as an intrinsic time-varying signal r(t) and spikes are seen as random events occurring at rate r(t). In all these definitions, rate is an abstract quantity defined on the spike trains. Therefore when stating that neural computation is based on rates rather than spike timing, what is meant is that the concept of rate captures most of the important details of neural activity and computation, while precise spike timing is essentially meaningless. On the other hand, when stating that spike timing matters, it is not meant that rate is meaningless; it simply means that rate is not *sufficient* to describe neural activity. Thus, these are not two symmetrical views: the stronger assumptions are on the side of the rate-based view. Of course, each *specific* spike-based theory makes a number of possibly strong assumptions. But the general idea that neural dynamics is based on individual spikes and not just rates is a relatively weak assumption. The rate-based view is based on an approximation, and the question is whether this is a good one or a bad one.

### Rate in Spike-Based Theories

The confusion in the assertion that “both rate and spike timing are important for coding” stems from the use of the word “coding”. Rates and spikes exist and vary with stimuli in both rate-based and spike-based theories. In either type of theory, rate and spike timing both “encode” stimuli, in the sense of information theory, and therefore the coding perspective is generally not the right way to distinguish between those theories—with a few exceptions when it can be shown that rates are not sufficiently informative about stimuli to account for behavior (Jacobs et al., 2009).

Specifically, the spike-based view does not in itself deny the importance of the firing rate, it only denies its status as the basis of computation. What do spike-based theories have to say about firing rate? First of all, rate is important in spike-based theories. The timing of a spike can only exist if there is a spike. Therefore, the firing rate generally determines the rate of information in spike-based theories, but it does not determine the content of information. For example, in Denève’s predictive coding theory (Boerlin et al., 2013) and more generally in spike-based coding theories (e.g., Smith and Lewicki, 2006), neurons spike when spiking at that time reduces an error criterion defined on spike trains. Thus the firing rate correlates with the error signal, but the corrective signal is carried by the precise timing of spikes.

A very important concept in sensory neuroscience is the selectivity curve or tuning curve, which measures how the firing rate of a neuron varies with one particular aspect of stimuli. For example, many cells in the primary visual cortex (V1) fire more in response to a moving bar or grating with a specific orientation (Hubel and Wiesel, 1959). The firing rate could then be said to “encode” the orientation of bars. But the level of oxygen in the blood also varies with orientation (Yacoub et al., 2008), in a similar way, and so it can also be said that oxygen level “encodes” the orientation of bars. Can we conclude that the basic element of computation in the brain is blood oxygen level? Clearly, the fact that an observable co-varies with stimulus parameters does not in itself imply that the observable has any causal role in processing the stimulus.

Tuning curves of V1 neurons may form the basis of orientation processing in the visual system, or they may be a correlate of orientation processing—or more generally, a correlate of processes that depend on orientation. Specifically, any spike-based theory in which spiking incurs a cost (as in e.g., Boerlin et al., 2013) predicts that the firing rate covaries with the stimulus parameters involved in the processing, and therefore that the rate “encodes” those parameters to some extent. The firing rate then represents energy consumption (Attwell and Laughlin, 2001), not computation.

From these observations, it follows that, in spike-based theories, firing rate is a *correlate* of information processing in a neuron. This stands in contrast with rate-based theories, in which rate is the *basis* of information processing. But both types of theories predict that firing rates correlate with various aspects of stimuli—and therefore that there is information about stimuli in firing rates for an external observer. Therefore, the fact that firing rates vary in a systematical way with various aspects of stimuli is consistent with both views. The difference between the two types of theories is that in spike-based theories the firing rate measures the *quantity* of information (energy consumption), while in rate-based theories it constitutes the *content* of information.

Therefore the question is not whether firing rate or spike timing matters or is informative about external stimuli, but about which one is the basis of computation. In broader terms, the question is whether the firing rate has a causal role in the dynamics of the system.

## Assertion #2: Neural Responses are Variable, therefore Neural Codes can Only be Based on Rates

Perhaps the most used argument against spike-based theories is the fact that spike trains *in vivo* are variable both temporally and over trials (Shadlen and Newsome, 1998), and yet this might well be the least relevant argument. This assertion is what philosophers call a “category error”, when things of one kind are presented as if they belonged to another. Specifically, it presents the question as if it were about variability vs. reproducibility. I will explain how variability can arise in spike-based theories, but first an important point to make is that the rate-based view does not *explain* variability, but rather it simply states that there is variability.

### The Variability Argument

**Figure 2:**
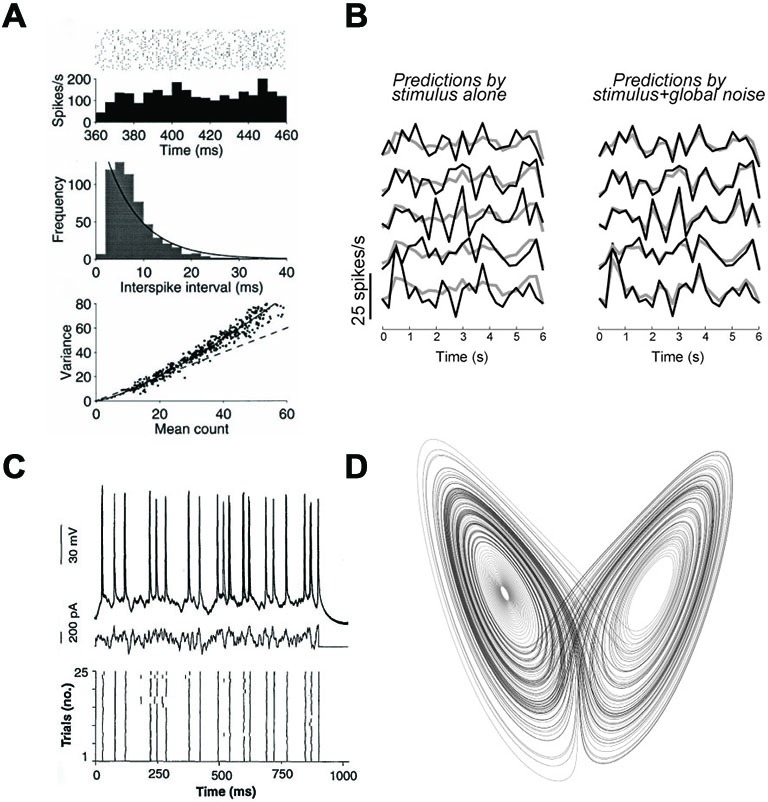
**Neural variability. (A)** Responses of a MT neuron to the same stimulus (reproduced from Shadlen and Newsome, 1998). Top: spike trains over repeated trials, with the corresponding firing rate, meant as a firing probability (Figure 1C). Middle: distribution of Interspike intervals (ISIs), with an exponential fit (solid curve). Bottom: variance of spike count as a function of mean count, with the prediction for Poisson processes (dashed). **(B)** Responses of the same V1 neuron over five trials of the same stimulus, represented as temporal firing rate (adapted from Schölvinck et al., 2015). Left: comparison with the average response (gray curve), showing variability over trials. Right: comparison with a prediction using the responses of other neurons, showing that the variability does not reflect private noise. **(C)** Responses of a single cortical neuron to a fluctuating current (middle) injected *in vitro* (reproduced from Mainen and Sejnowski, 1995). Top: superimposed voltage traces. Bottom: spike trains produced in the 25 trials. **(D)** The Lorentz attractor, consisting of trajectories of a chaotic three-dimensional climate model. Chaos is not randomness, as it implies particular relations between the variables represented by the attractor.

There are two ways to understand the term “variable” and I will first discard the meaning based on temporal variability. Interspike intervals (ISIs) are highly variable in the cortex (Softky and Koch, 1993), and their distribution is close to an exponential (or Gamma) function, as for Poisson processes (possibly with a refractory period; Figure 2A). This could be interpreted as the sign that spike trains are realizations of random point processes. This argument is very weak, because the exponential distribution is also the distribution with maximum entropy for a given average rate, which means that maximizing the information content in the timing of spikes of a single train also implies an exponential distribution of ISIs (Rieke et al., 1999). Temporal variability cannot distinguish between rate-based and spike-based theories, even in terms of coding.

Therefore the only reasonable variability-based argument in support of the rate-based view is the variability of spike trains across trials, that is, the lack of reproducibility. In the cortex (but not so much in some early sensory areas such as the retina (Berry et al., 1997) and some parts of the auditory brainstem (Joris et al., 1994)), both the timing and number of spikes produced by a neuron in response to a given stimulus varies from one trial to another (Shadlen and Newsome, 1998). This means that the response of a neuron to a stimulus cannot be described by a deterministic function of that stimulus. It could be stochastic, chaotic, underdetermined, or dependent on an uncontrolled variable (e.g., attentional state). This is the only fact that such observations tell us. In particular, it does not tell us that neural variability in the brain necessarily results from random spiking processes with rates defined by deterministic continuous dynamics, i.e., the rate-based view. The next sections will provide examples of processes that do not follow this scheme.

Therefore, the argument of spike train variability is about reproducibility, not about rate-based vs. spike-based theories. In principle, it can only discard a deterministic spike-based theory based on absolute spike timing, that is, requiring reproducible spike timing with respect to the stimulus. However, spike-based theories are generally based on relative timing across different neurons (for example synchrony (Abeles, 1991; Izhikevich, 2006; Brette, 2012) or rank order (Thorpe et al., 2001)), not on absolute timing.

In fact, the argument can be returned against rate-based theories. The use of this argument seems to imply that rate-based theories take into account biological variability, whereas spike-based theories do not. But in fact, quite the opposite is true. Rate-based theories are fundamentally deterministic, and a deterministic description is obtained at the cost of averaging noisy responses over many neurons, or over a long integration time (for example “neural mass” or “mean field” models; Deco et al., 2008). On the other hand, spike-based theories take into account individual spikes, and therefore do not rely on averaging. In other words, it is not that rate-based descriptions account for more observed variability, it is just that they *acknowledge* that neural responses are noisy, but they do not account for any variability at all. This confusion may stem from the fact that spike-based theories are often described in deterministic terms. But as stressed above, rate-based theories are *also* described in deterministic terms.

The question is not whether spikes are reproducible; it is whether the spiking interactions of neurons can be reduced to the dynamics of average rates, in the same way as the mechanics of individual particles can be reduced in some cases to the laws of thermodynamics. This possibility does not follow at all from the observation that the response of a given neuron is not the same in all trials. In other words, the observation of variability itself says little about the nature of the process that gives rise to that variability. As I will now describe in more detail, a deterministic spike-based theory can be consistent with variability due to a variety of causes, in particular: state-dependence, deterministic chaos, degeneracy.

### State-Dependence

When a given sensory stimulus is presented several times, the same neuron often responds differently over repeated trials. However, we mean “the same” neuron in an anatomical sense, in the same way as we would speak of “the same” organism. But the very fact that we think and behave also means that we are never exactly in the same state at any instant. Neurons might be anatomically stable, but their state is not. Therefore, the observation of non-reproducibility of neural responses may simply reflect the fact that the state of the neuron or of the network it is embedded in differs between trials (Masquelier, 2013; Renart and Machens, 2014).

First, many neurons, in particular cortical neurons, are spontaneously active, that is, are active in the absence of any sensory stimulus. This simple fact implies that those neurons will almost never be in the same state over repeated trials, and this activity can account for a large part of observed variability (Arieli et al., 1996). In fact, it has been shown both in the visual cortex (Schölvinck et al., 2015) and in the auditory cortex (Deweese and Zador, 2004) that a large part of the variability of neural responses to stimuli can be accounted for by the variability in the activity of the neighboring network, rather than by private variability intrinsic to the recorded neuron. For example, Figure 2B (from Schölvinck et al., 2015) shows that the responses of a neuron, which vary between trials, can be well predicted by its trial-averaged response (gray) plus a term proportional to the sum of deviations from the mean responses of the other neurons, observed in the same trial (termed “global noise”). In other words, a large part of inter-trial variability seems to reflect global modulation of the responses of all neurons.

How much of this stimulus-unlocked activity is “noise”? Certainly, not all of it. A basic anecdotal remark is that, in humans, consciousness does not vanish when external stimulation stops. At a more physiological level, it is also known that spontaneous activity is structured (Luczak et al., 2007) and influenced by previous stimulus-driven activity (Bermudez Contreras et al., 2013). An influential theory, predictive coding theory, proposes that responses of neurons to sensory stimuli reflect the combination of a feedforward stimulus-driven input with a prediction mediated by higher order areas (Rao and Ballard, 1999). If the prediction depends on previous sensory experience, then it follows that responses to the same repeated stimulus would vary over trials. Here variability reflects the internal change in sensory prediction, not noise (Berkes et al., 2011). This interpretation is in line with the general notion that biological systems are anticipatory systems (Rosen, 1985), and more generally with the notion that behavioral responses depend not only on the presented sensory stimulus but also on memory. This view is supported by several studies showing that behavioral variability is partly due to the influence of the recent history of stimulus presentations (Gold et al., 2008; Marcos et al., 2013; Raviv et al., 2014).

Second, at the single neuron level, as observed in a slice, responses are much more reproducible than when the neuron is embedded in an active network (Figure 2C). Specifically, the responses of neurons to fluctuating currents injected at the soma are reproducible at the millisecond timescale (Bryant and Segundo, 1976; Mainen and Sejnowski, 1995). In addition, dynamic photostimulation of presynaptic neurons also results in reliable responses in cortical neurons, which means that synaptic transmission and dendritic processing contribute a small amount of noise, possibly because of multiple synaptic contacts between cells (Nawrot et al., 2009). Reproducible responses are observed despite the fact that the state of a neuron also changes over trials in those experiments. In particular, adaptation in neurons has power law characteristics, meaning that they adapt on all time scales (Lundstrom et al., 2008). Therefore, despite the experimental overestimation of noise, *in vitro* experiments show that intrinsic neural noise is generally low.

In summary, the lack of reproducibility of neural responses to sensory stimuli does not imply that neurons respond randomly to those stimuli. There are a number of sensible arguments supporting the hypothesis that a large part of this variability reflects changes in the state of the neuron or of its neighbors, changes that are functionally meaningful. This comes in addition to the remark that stochasticity does not imply that the dynamics of neural networks can be reduced to the dynamics of average rates.

### The Chaos Argument

A counter-argument to the idea that variability might be due to uncontrolled but deterministic processes is that a large part of the observed neural variability is irreducible because neural networks are chaotic, that is, they are sensitive to initial conditions (van Vreeswijk and Sompolinsky, 1996; Banerjee et al., 2007; London et al., 2010). Indeed, if neural networks are chaotic, then their responses would still not be reproducible even if all stimulus-unrelated variables were controlled (e.g., attention or memory). However, the argument misses its target because the idea that rates entirely capture the state of the system does not follow from lack of reproducibility.

In a chaotic system, nearby trajectories quickly diverge. This means that it is not possible to predict the state of the system in the distant future from the present state, because any uncertainty in estimating the present state will result in large changes in predicted future state. For this reason, the state of the system at a distant time in the future can be seen as stochastic, even though the system itself is deterministic. Specifically, while *in vitro* experiments suggest that individual neurons are essentially deterministic devices (Mainen and Sejnowski, 1995), a system composed of interacting neurons can be chaotic, and therefore for all practical aspects their state can be seen as random, so the chaos argument goes.

The fallacy of this argument comes from the common confusion between deterministic chaos and randomness. There are at least two important well-known differences between chaos and randomness (see a textbook on chaos theory for more detail, e.g., Alligood et al., 1997). One is *recurrence*, that is, the fact that similar short-term trajectories can reappear, although at possibly unpredictable times. Recurrence follows trivially from the fact that the system is deterministic: similar states will produce similar trajectories in the short run, even though they might ultimately diverge. In the prototypical chaotic system, climate, it is well known that the weather cannot be accurately predicted more than 15 days in the future, because even tiny uncertainties in measurements make the climate models diverge very quickly. However, it is still possible to make relatively accurate short-term predictions over a few days, because a given atmospheric configuration can lead to a predictable sequence of climatic events. For example, a rapid drop in barometric pressure is often followed by rain.

Recurrence is an explicitly postulated property of neural networks in some spike-based theories, for example synfire chains (Diesmann et al., 1999; Ikegaya et al., 2004) and polychronization, which is an extension of synfire chains to spatiotemporal patterns of spikes (Izhikevich, 2006; Szatmáry and Izhikevich, 2010). Neither theory requires reproducibility of spike timing, and indeed models that have been shown to instantiate those theories include either background noise (Diesmann et al., 1999) or background network activity (Szatmáry and Izhikevich, 2010). Those theories only rely on the possibility of recurring patterns, which is compatible with deterministic chaos.

In addition, other spike-based theories do not focus on recurrence but do not require long-term predictability either. All theories based on coincidence detection require stable relative timing on the time scale of a neuron’s integration window, which does not exceed a few tens of ms. Denève’s predictive coding theory critically relies on relative spike timing but does not require reproducible spiking patterns (Boerlin et al., 2013).

The second important difference between deterministic chaos and randomness has to do with relations between variables. According to the chaos argument, because precise spikes are not reproducible, they can be equivalently replaced by random spikes with statistics (rates) given by their long-term distributions. This inference is incorrect in the case of deterministic chaos. Taking the case of climate again, a counter-example is the Lorenz system, a chaotic system of three differential equations representing the evolving state of a model of atmostpheric convection. The abovementioned argument would mean that the behavior of the system can be adequately captured by replacing the state variables by their long-term distributions. Even if we allowed correlations between those variables, this would mean that trajectories of the system fill a three-dimensional manifold. Instead, trajectories lie in a lower-dimensional manifold called *strange attractor* (Figure 2D), meaning that state variables are more constrained than implied a continuous three-dimensional distribution (e.g., a multivariate Gaussian distribution). In terms of spiking networks, this means that the behavior of a chaotic spiking network cannot be captured by a rate-based description.

In fact, these major differences between deterministic chaos and randomness imply that the chaos argument is an argument *against* rate-based theories, precisely because a chaotic system is *not* a random system. Specifically, deterministic chaos implies: (1) short-term predictability of spike trains; (2) recurrence of precise spike patterns, and most importantly; and (3) insufficiency of rate-based descriptions.

### Degeneracy

**Figure 3:**
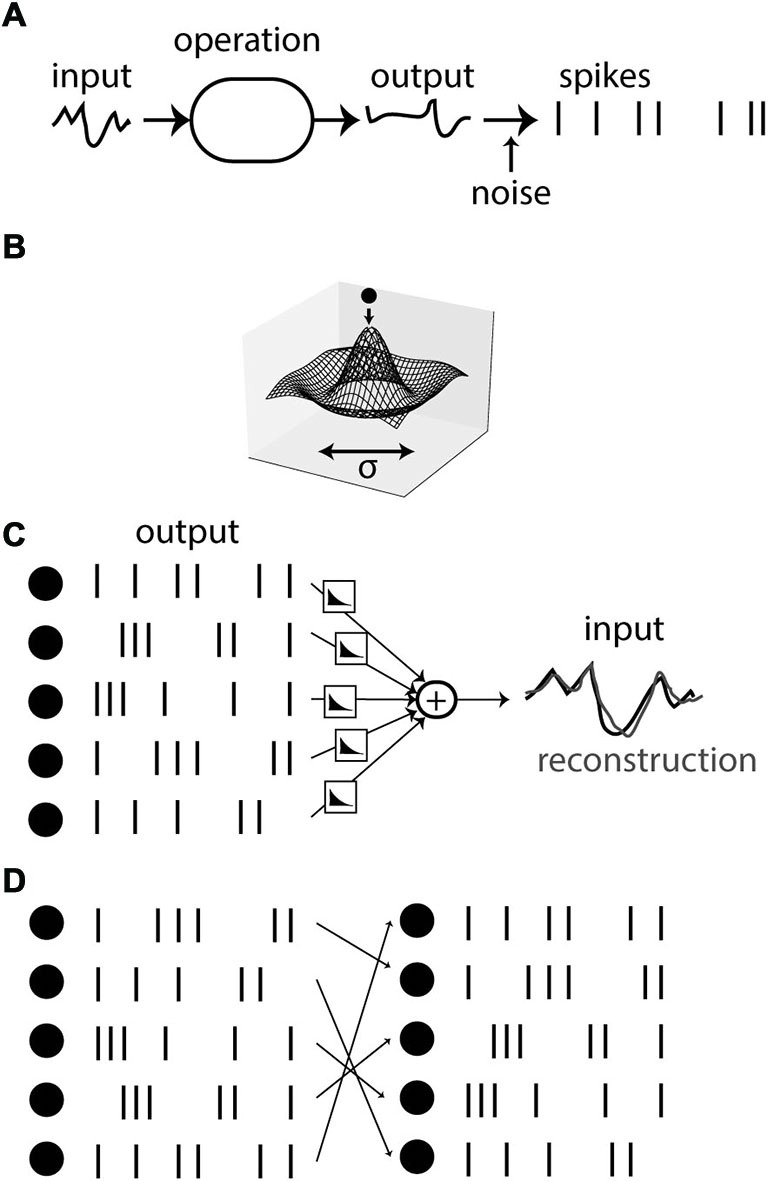
**Variability due to degeneracy. (A)** Spikes can be seen as the result of a sequence of operations applied on an input signal, followed by spike generation. In this view, variability comes from noise added in the spiking process. **(B)** The state of a physical system can often be described as a minimum of energy. Symmetries in the energy landscape can imply observed variability, whose magnitude bears no relation with the amount of intrinsic noise. **(C)** An example of the energy view is spike-based sparse coding. A reconstruction of the signal is obtained from combining filtered spike trains together, and spikes are timed so as to make the reconstruction accurate. **(D)** If the system is redundant, the reconstruction problem is degenerate, leading to several equally accurate spiking solutions (here obtained by permutation of neurons).

Finally, variability can also arise in deterministic systems when neural responses are underconstrained by the stimulus. Underlying the argument of neural variability is the assumption that spikes are produced by applying some operation on the stimulus and then producing the spikes (with some decision threshold; Figure 3A). The variability of spike timing between trials, so the argument goes, must then reflect a corresponding amount of noise, inserted at some point in the operation. However, the observed state of a physical of system can often be understood in a different way, as the state minimizing some energy (Figure 3B). If the energy landscape has symmetries, then different states have the same energy level and therefore have the same probability of being observed. In the case of the Mexican-hat energy landscape shown on Figure 3B, any state on the low energy circle may be observed. This property of physical systems is called *degeneracy*. Although in a Newtonian view, the existence of this variability may be ultimately due to variations in initial state or intrinsic noise, the amount of observed variability is determined by the structure of the energy landscape, not by the amount of intrinsic noise, which could be infinitesimal. In addition, the observed variability is highly structured: in this case, states lie on a particular circle—note that this implies a highly constrained relation between the two observables even though linear correlation is null.

Some spike-based theories follow the energy-minimization view. An example is provided by the theory of sparse coding (Olshausen and Field, 2004) applied to spikes. It has been used for example to explain the receptive field of auditory neurons (Smith and Lewicki, 2006), and recently it was related to the dynamics of spiking neurons in an asynchronous spike-based theory (Boerlin et al., 2013). In this theory, it is postulated that the time-varying stimulus can be reconstructed from the firing of neurons, in the sense that each spike contributes a “kernel” to the reconstruction, at the time of the spike, and all such contributions are added together so that the reconstruction is as close as possible to the original stimulus (Figure 3C). Note how this principle is in some way the converse of the principle described in Figure 3A: spikes are not described as the result of a function applied to the stimulus, but rather the stimulus is described as a function of the spikes. Thus spike encoding is defined as an inverse problem rather than a forward problem. This approach has been applied to the retina, where it was shown that the position of a moving bar can be accurately reconstructed from the firing of ganglion cells (Marre et al., 2014). In the theory of Denève and colleagues (Boerlin et al., 2013), neurons fire so as to reduce the spike-based reconstruction error; that is, the membrane potential is seen as a reconstruction error and the threshold as a decision criterion.

An interesting point with regard to the issue of neural variability is that, because the pattern of spikes is seen as a solution to an inverse problem, there can be sensory stimuli that are consistent with several patterns of spikes (Figure 3D). That is, the reconstruction problem can be degenerate, if there are regularities in the stimulus or redundancies in what neurons encode (kernels). In the energy view, this means that there are several states with the same energy level (energy being stimulus reconstruction error). Imagine for example that two neurons contribute exactly the same kernel to the reconstruction. Then on one given trial, either of these two neurons may spike, perhaps depending on tiny differences in their current state, or on the random switch of a ionic channel. From the observer point of view, this represents a lack of reproducibility. However, this lack of reproducibility is precisely due to the precise spike-based coordination between neurons: to minimize the reconstruction error, exactly one of the two neurons should be active, and the timing should be precise too. In contrast with rate-based theories, the idea of spike-based coordination (i.e., optimal placement of spikes so as to minimize some energy) predicts that reproducibility should depend on properties of the stimulus, in particular on some notion of regularity. Here the observed of reproducibility bears no relation with the precision of the spike-based representation, which, by construction, is optimal.

To summarize this set of points, the observation of neural variability in itself says little about the origin of that variability. In particular, variability in individual neural responses does not necessarily reflect private noise. Generally, any theory that does not see the sensory responses of neurons as an essentially feedforward process predicts a lack of reproducibility. Thus the existence of neural variability does not support the rate-based view. In fact, any successful attempt to explain the origin of that variability undermines rate-based theories, because the essence of the rate-based view is precisely to explain neural variability away by modeling it as private noise.

## Assertion #3: the Difference Between Rate-Based and Spike-Based Theories is a Question of Timescale

It is tempting to view the difference between rate-based and spike-based theories as one of the timescale of the description (short timescale for spike-based theories, long timescale for rate-based theories). This misconception again stems from a confusion between coding, which is about relating stimulus and neural activity for an external observer, and computation (in a broad sense), which is about the way neurons interact with each other.

**Figure 4:**
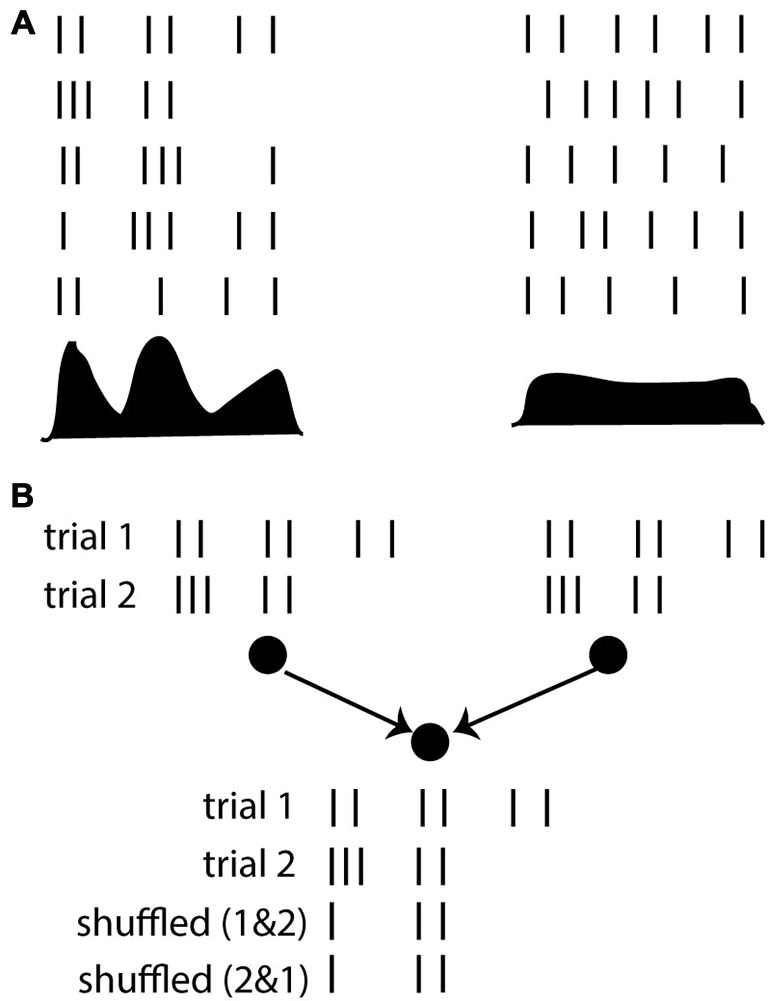
**The timescale argument. (A)** Responses of a neuron over repeated trials, where the firing rate (PSTH shown below) varies on a fast (left) or slow (right) time scale. **(B)** Whether the firing rate varies quickly or slowly, the average rate is not generally sufficient to predict the response of a postsynaptic neuron. Here the responses of two neurons are shown over two trials. The postsynaptic neuron responds strongly when the presynaptic spike trains are taken in the same trial (because they are synchronous), but not if the spike trains are shuffled over trials.

One may for example consider the response of a neuron to a stimulus over repeated trials and measure its post-stimulus time histogram (PSTH). It seems that if the PSTH is peaky then we should talk of a “spike timing code” and if it changes more gradually a “rate code” might seem more appropriate, but really these are words to describe the more accurate description, which is the PSTH itself, with its temporal variations (Figure 4A). That is, considering neuron firing as a point process with a time-varying rate given by the PSTH is as good a description as it gets.

The fallacy of this argument lies in the choice of considering neural responses exclusively from the point of view of an external observer (the coding perspective), entirely neglecting the interactions between neurons. It may be correct that the PSTH provides a good statistical description of input-output responses of that neuron. But on any given trial, neurons do not deal with PSTHs. They deal with spike trains. On a given trial, the firing of a given neuron is* a priori* determined by the spike trains of its presynaptic neurons, not by their PSTHs. There is no guarantee that the (time-varying) rate of a neuron can be described as a function of presynaptic rates. If it were the case, then a neuron would respond identically if the presynaptic spike trains were shuffled over trials, but it is trivial to show cases when this is not true (Figure 4B). In any nonlinear system, the average output is generally not a function of the average inputs, and making such an assumption has little to do with the description timescale.

**Figure 5:**
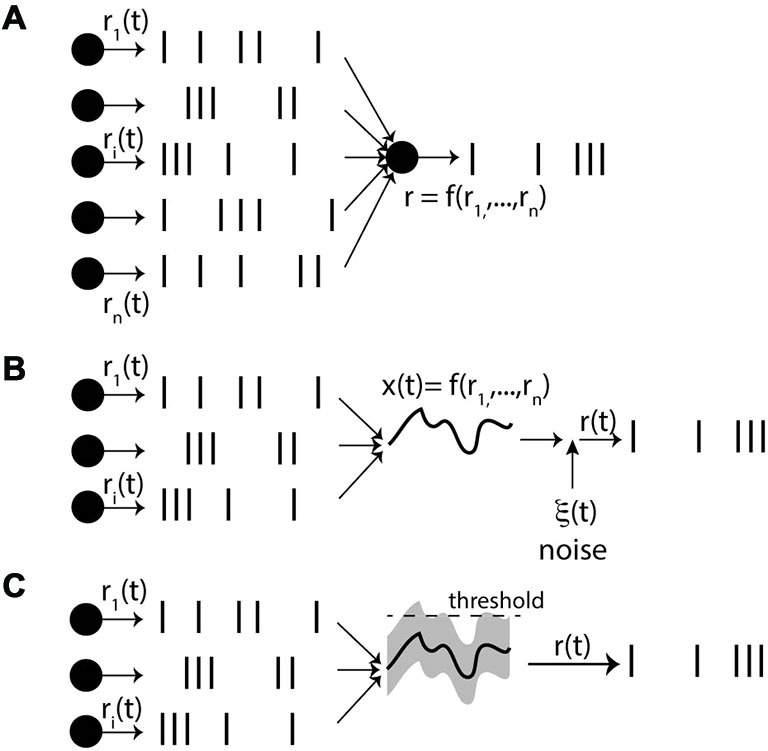
**The rate-based view. (A)** Each neuron is described by a private quantity r(t), its rate, which determines the spike trains through a stochastic process. Crucially, even though only the spike trains are directly observed by the neuron, it is postulated that the rate r(t) only depends on the presynaptic rates r_i_(t), and not on the spike trains themselves (*f* can be a function, dynamical process or filter). **(B)** One option is that synaptic integration leads to a quantity x(t); (e.g., membrane potential) that only depends on the rates r_i_(t) by law of large numbers. As x(t) is deterministic, private noise must be added so as to produce stochastic spike trains. **(C)** The other option is that there are random fluctuations around the mean x(t) due to the spiking inputs, causing output spikes. For these fluctuations to depend only on the rates, the presynaptic processes must be independent, but we note that we have now introduced correlations between inputs and outputs.

We can now start to spell out the rate-based view (Figure 5A). It is postulated that: (1) for every neuron there exists a private quantity r(t); (2) spike trains are produced by some random point process with rate r(t); and (3) the rate r(t) of a neuron only depends on its presynaptic rates r_i_(t), according to a dynamical process. The really problematic postulate here is the third one. The neuron does not have direct access to the presynaptic rates: it has indirect access to them through the spike trains, which are specific realizations of the processes. Thus this assumption means that the operation performed on input spike trains, which leads to the rate r(t), is essentially independent of the specific realizations of the random processes.

How can assumption (3) be satisfied? One possibility that comes to mind is the law of large numbers—this is the essence of “mean-field” approaches (Figure 5B). If it can be applied, then integrating inputs produces a deterministic value that depends on the presynaptic rates, independent of higher statistical orders (e.g., variance). But then the source of noise in the spiking process (2), which produces stochastic spike trains from a deterministic quantity, must be entirely intrinsic to the neuron (see e.g., Ostojic and Brunel, 2011). Given that experiments *in vitro* suggest that intrinsic noise is very low (Mainen and Sejnowski, 1995), this is a fairly strong assumption. The other alternative is that random spikes are produced by random fluctuations of the total input around its mean (higher statistical orders; Figure 5C). But for these fluctuations to depend only on the presynaptic rates, the inputs must be independent. Here, we arrive at a critical difficulty, because we have just introduced correlations between inputs and outputs, by allowing output spikes to be produced by fluctuations in the total input. Therefore, the required assumption of independence will not generally hold when the process is repeated over several layers, or when neurons are recurrently connected. One way of addressing this difficulty is to postulate that neurons are randomly connected with small probability, so that presynaptic neurons are effectively independent, in addition to a large amount of private noise (Brunel, 2000), which I will comment in more detail in “Conclusion” Section.

What this discussion shows is that the problem has little to do with the notion of timescale. It has to do with whether it is possible to come up with a consistent model of neural activity in which rate is a causal variable, in a way that is consistent with biophysics. As it turns out, this is not a trivial task.

## The Important Question

Does the brain use a rate code or a spike timing code? The conclusion of this analysis is that the phrasing of this question is unfortunate. It casts the problem in the perspective of coding, that is, as the problem of the relationship between external stimuli and particular observables (rate or spike timing). It entirely misses the core of the problem, which is to know whether those observables have a causal role in the activity of the system, or rather are a correlate of that activity. From that perspective follow several misconceptions, in particular the idea that neural variability is a challenge to spike-based views, and the idea that the problem is about the timescale of description. It should be stressed that none of the arguments I have listed against spike-based views have any logical validity, because they address the wrong question.

What is the right question then? We are considering two types of models of the brain, spike-based models and rate-based models. What is a model? A model is a formal system that consists of observables and inference rules (see Rosen’s (1985) excellent treatment of this question). It is a model of a particular natural system when two properties are satisfied: (1) the natural system can (potentially) be “encoded” into observables of the model by means of measurement and (2) causality in the natural system corresponds to inference in the model, that is, the evolution of the natural system maps to changes in observables that are consistent with the inference rules. This definition can be made more general, but is sufficient for our purpose.

In the spike-based view, the observables are spike trains. Spikes produce instantaneous changes in state variables of neurons, which may or may not be observables [e.g., the membrane potential as in the Hodgkin-Huxley model or some more abstract variable as in the quadratic model (Ermentrout, 1996)]. These variables typically evolve according to some continuous dynamical system (deterministic or stochastic), which usually follows from biophysical considerations. Spikes are then produced and communicated to other neurons through some form of decision on the neuron’s state variables. Thus, generally, a spike-based model defines the relation between input spike trains and output spike trains. It predicts future spikes from the past history of spikes. There are a wide variety of spike-based theories, which differ by the particular relations that are assumed between spikes.

In the rate-based view, the observables are rates, which are defined as some form of average over spikes. A rate-based model then defines relationships between those rates over time, typically taking the form of a continuous dynamical system (e.g., a set of differential equations).

**Figure 6:**
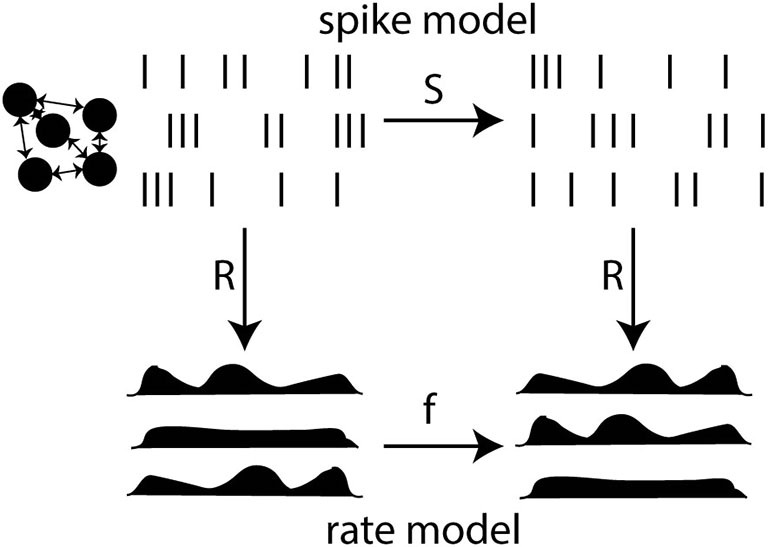
**Reduction of a spike-based model to a rate-based model.** The spike model defines a relationship between presynaptic and postsynaptic spike trains through a function S. The rate model defines a relationship between rates through a function f. Rates are related to spikes through an observation function R. Reduction is possible when the diagram commutes: the composition of R and S equals the composition of f and R (mathematically, R o S = f o R). This is not generally possible because R is not invertible (many spike trains have the same rate).

The key point is that rates are not independent observables. They are defined from spikes. The rate-based view does not refute the idea that spikes produced by a neuron depend on presynaptic spikes through some integration process. Rather, it postulates that spike-based dynamics can be approximated by a rate-based model. In other words, the rate-based postulate is the postulate that, under appropriate assumptions, a spike-based model can be reduced to a rate-based model (Figure 6). This means that, when spikes are mapped to rates (or conversely), inference in the spike-based model is mapped to inference in the rate-based model. To back up this postulate means two things: (1) to determine under which assumptions this formal reduction is possible and (2) to test whether those assumptions are empirically valid. It should be noted that a large part of this problem is therefore of theoretical nature.

Thus, a possible way to rephrase the question is the following: *is it possible to reduce the spiking interactions of neurons to the interaction of rates?* Where reduction is meant in the sense of model reduction.

There is a striking analogy between this problem and the articulation between thermodynamics and mechanics. Indeed there are two ways to model a gas. A macroscopic analysis, in which macroscopic observables (pressure, volume, temperature) are related to each other, and a microscopic analysis, in which individual particles are modeled by Newtonian mechanics. An entire field of physics, statistical mechanics, is motivated by the goal of making these two views consistent. Under some conditions, the mechanical model can be reduced to some extent to the thermodynamical model, and it is fair to say that this reduction is far from trivial. The question at hand regarding spike-based and rate-based views is of this kind: can the spiking interactions of neurons be captured by some form of thermodynamical model?

It is important to highlight a few important differences with thermodynamics. The first point to note is that classical thermodynamics was not initially derived from mechanics. Rather, the laws of thermodynamics were established independently of any mechanical description, and only later was the connection attempted. In contrast, while there are relatively well-established biophysical models of spike-based interactions, no laws of this kind have so far been empirically established at a macroscopic level, that is, in terms of rate-based interactions, and it is an open question that such laws can even be formulated. The second point is that the reduction of mechanics to thermodynamics requires some sort of homogeneity between particles, that is, that all particles play essentially the same role in the macroscopic object. Applied to neurons, this is a very strong requirement, especially as the structure of neural networks is shaped by activity-dependent plasticity, resulting in particular from the interaction of the organism with the environment, and not only by random factors.

Thus, at the very least, there appears to be no obvious reason why spiking interactions could be reduced to the continuous dynamics of rates. But now that the question has been rephrased in a more meaningful way, I will provide a few elements of answer.

## Can the Dynamics of Spiking Networks be Reduced to Rates?

The general strategy to determine whether a formal reduction of a spike-based model to a rate-based model is possible is as follows. The first step is to derive the rate-based model that is consistent with the spike-based model. We start by specifying the relation between rates and spikes, for example we assume that spike trains are independent realizations of Poisson processes with rates r_i_(t). We then apply the inference rules of the spike-based model, for example we calculate the properties of spike trains produced by a spike-based neuron model receiving Poisson inputs. In particular, we obtain a relation between input and output rates, which is the inference rule in the rate-based model imported from the spike-based model (see for example, Ostojic and Brunel, 2011).

The second step is to examine whether and under what conditions those properties conform to the initial assumption, for example that output spike trains are close to independent Poisson processes. They may be drastically different, in which case one may look either for a different relation between spikes and rates, or for a different spike-based model. Spike trains produced by the spike-based model will never exactly conform to the rate-based assumption, for the simple reason that inputs and outputs cannot be statistically independent, since the former determine the latter.

The third step is thus to check whether the violation of the rate-based assumption is strong enough to introduce systematic deviations between the dynamics of the two models. For example, for a recurrent network, the derived inference rule is used to determine self-consistent rates, and we can then check whether these rates match those observed in the spike-based model (for example by numerical simulation). In the same way, in a multilayer feedforward neural network, one can check that the repeated application of the rate-based operation over successive layers yields the same output rates as those observed in the spike-based model, where deviations might have been introduced in the intermediate layers.

### The Fluctuation-Driven Regime

In the 1990s, this strategy was used in a famous published exchange about the rate vs. timing debate. Softky and Koch (1993) argued that if spike trains were random (independent Poisson processes), as they seemed to be in single unit recordings, and if cortical neurons sum many inputs (about 10,000 synapses for a pyramidal cell), then by the law of large numbers their output should be regular, since the total input would be approximately constant. Therefore, so they argued, there is an inconsistency in the two hypotheses (independence of inputs and integration). They proposed to resolve it by postulating that neurons do not sum their inputs but rather detect coincidences at a millisecond timescale, using dendritic nonlinearities. Shadlen and Newsome (1994) demonstrated that the two hypotheses are in fact not contradictory, if one postulates that the total mean input is subthreshold, so that spikes only occur when the total input fluctuates above its average. This is called the “fluctuation-driven regime”. An electrophysiological signature of this regime is a distribution of membrane potential that peaks well below threshold (instead of monotonically increasing towards threshold, as in the mean-driven regime), for which there is experimental evidence (see Figure 7 of Rossant et al., 2011; apparent diversity is due to the presence of up and down states in anesthetized preparations). When there are many inputs, this can happen when excitation is balanced by inhibition, hence the other standard name “balanced regime” (note that balanced implies fluctuation-driven, but not the other way round).

In the fluctuation-driven regime, output spikes occur irregularly, because the neuron only spikes when there is a fluctuation of the summed input. Thus the two hypotheses (random Poisson inputs and irregular output firing) are not contradictory: it is completely possible that a neuron receives independent Poisson inputs, integrates them, and fires in a quasi-Poisson way, without any need for submillisecond coincidence detection. Thus, for a single neuron, it is possible to come up with a rate-based model that seems adequate. But note that the single neuron case is not a particularly challenging situation, for by assumption the output rate is a function of the input rates, since those are the only parameters of the inputs in this scenario. The significant result here is that, under certain conditions that seem physiologically plausible, the output will also be quasi-Poisson, in particular irregular.

Problems start when we consider that the neuron may be embedded in a network. As Softky (1995) correctly argued in response, output spikes are still determined by input spikes, so they cannot be seen as random. In fact, it is precisely in the fluctuation-driven regime that neurons tend to have precisely timed responses (Mainen and Sejnowski, 1995; Brette and Guigon, 2003). Specifically: input spike trains are independent Poisson processes, the output spike train is (approximately) a Poisson process, but inputs and outputs are not independent. The question is whether the dependence between an input and an output is weak enough that it can be ignored in the context of a network. If we assume that inputs and outputs have about the same average firing rate and there are N excitatory inputs, then there should be one output spike for N input spikes, and therefore the correlation between a given input and the output should be of order 1/N on the time scale of integration. How significant is 1/N for a pairwise correlation?

It turns out that the fluctuation-driven regime, which is necessary to preserve the statistical properties of Poisson processes, is also the regime in which neurons are most sensitive to correlations (Abeles, 1982; Rossant et al., 2011). How big should pairwise correlations be to have an impact on the output rate of a neuron? The answer is: about 1/N in the fluctuation-driven regime. It follows from a simple argument. In the fluctuation-driven regime, the output rate depends not only on the mean input but also on its variance (for otherwise the neuron would not fire at all). The variance consists of two terms: the sum of variances of each synaptic input, of order N, and the sum of covariances of all pairs of synaptic inputs, of order cN^2^, where c is the pairwise correlation. Thus the second term starts to have an impact as soon as pairwise correlations are of order 1/N. Higher-order correlations have even greater impact.

In other words, in the fluctuation-driven regime, correlations between inputs and outputs have just the right magnitude to produce a significant impact on network dynamics. In passing, we note that correlations of order 1/N are considerably smaller than what is typically considered near zero in the experimental literature (Ecker et al., 2010; Renart et al., 2010), because measuring such small correlations would require very long recordings or very large populations.

Thus, in standard biophysical neuron models, the requirement that output spike trains are irregular leads to the fluctuation-driven regime, but it is precisely in that regime that neurons are most robust to noise and sensitive to correlations. Correlations introduced between inputs and outputs by the spiking process are sufficiently strong to impact target neurons. This means that the rate-based postulate fails without additional assumptions, which I will discuss below.

### Recurrent Networks

The formal reduction of spike-based network models to rate-based models has been investigated mostly in random networks, notably by Nicolas Brunel and colleagues, using methods from statistical mechanics (Brunel, 2000). It is possible to derive equations that describe the transformation between the input rates of independent spike trains and the output rate of an integrate-and-fire model. In a statistically homogeneous network, one can then deduce the stationary firing rate of the neurons, which can be compared to numerical simulations. The approach has also been applied to calculate self-sustained oscillations (time-varying firing rates) in such networks (Brunel and Hakim, 1999). As we have seen, the approach cannot work in general, and therefore additional assumptions are introduced. In this line of work, two typical assumptions are: a large amount of private noise (independent between neurons) and sparse connectivity (i.e., pairs of neurons are connected randomly with low probability). Private noise reduces input-output correlations, and sparse random connectivity ensures that input and output neurons have few targets in common. Results of simulations diverge from theory when the connection probability increases, meaning that the rate-based reduction does not work in those cases. Unfortunately, neither of the two additional assumptions introduced to make this reduction possible are empirically valid. In particular, real neural networks do not look like random sparse networks, for example they can be strongly connected locally, neurons can be bidirectionally connected or form clusters (Song et al., 2005; Perin et al., 2011).

Thus another type of assumption has been investigated recently, which applies to densely connected networks. Instead of postulating that anatomical correlations are negligible, it is hypothesized that correlations are actively canceled.

### Cancellation of Correlations

As I explained above, the spiking process introduces correlations between inputs and outputs, and therefore between neurons that have inputs in common. A potential problem for the dynamics of neural network models is that in a recurrent network, those correlations can build up and lead to very unnatural dynamical behaviors. This is a problem for spiking models, independently of whether they can be reduced to rate models. Recently, it was shown that it is possible to avoid this problem in dense networks under appropriate assumptions, when inhibition tracks excitation (Renart et al., 2010; Litwin-Kumar and Doiron, 2012). In that case, neurons fire irregularly and “asynchronously”, in the sense that average pairwise correlations are of order 1/N. Neurons have then been described as “effectively independent” (Renart et al., 2010) or with “negligible” correlations (Litwin-Kumar and Doiron, 2012), but there are important qualifications to make here. Such correlations are negligible for the problem at hand, which is to avoid runaway synchronous activity. But they are not negligible *in general*. First, as discussed above, correlations of order 1/N are sufficient to have a substantial impact on target neurons. Second, the 1/N scaling is a spatial and temporal signed average. The absolute magnitude of individual pairwise correlations is actually of order 1/√N, which is much larger (i.e., there are positive and negative correlations that cancel out when averaged over all pairs). This implies in particular that when the network structure is not uniform, correlations can be systematically larger in some parts of the network (Litwin-Kumar and Doiron, 2012). Third, correlations are temporal averages (by definition): two neurons can be weakly correlated and yet occasionally participate in events of synchronous firing within a group of neurons. One should not confuse synchrony, which is the transient simultaneous firing (at the integration time scale) of a group of neurons, and correlation, which a long-term probability of simultaneous firing of two neurons. Synchrony plays a central role in a number of spike-based theories (Abeles, 1991; Izhikevich, 2006; Szatmáry and Izhikevich, 2010; Brette, 2012) but correlations rarely do. For all these reasons, low correlations are not a sufficient condition to derive a rate-based model from a spike-based model.

To clarify the point that low correlations do not specifically support the rate-based view, I will now briefly discuss correlations in spike-based theories. Weak correlations are actually a wanted feature of all spike-based theories. This point was indeed acknowledged in the abovementioned study (Renart et al., 2010): “By preventing uncontrolled network-wide synchrony, this mechanism generates a background of weakly correlated spiking, as required for efficient information processing based on either firing rates or coordinated spike timing patterns”. This is indeed so even (and perhaps especially) in the spike-based theories that put most weight on synchrony, the theory of synfire chains (Griffith, 1963; Abeles, 1991) and its extensions (synfire braids (Bienenstock, 1995) and polychronization (Izhikevich, 2006; Szatmáry and Izhikevich, 2010)) and the theory of synchrony invariants (Brette, 2012). In the latter, for example, synchrony reflects the presence of some invariant structure in the sensory signals, that is, the property that the sensory signals follow some particular sensory law. It is therefore meant to be stimulus-specific. In all these theories, synchrony is a meaningful event and therefore, it must be rare. In this respect, all mechanisms that tend to cancel expected correlations (for example those due to anatomical factors) are desirable features. There are also spike-based theories that are based on asynchrony (Thorpe et al., 2001; Boerlin et al., 2013), for which low correlations are a basic feature.

## Conclusions

Rate-based and spike-based theories of the brain have been often compared to each other by addressing the following question: “does the brain use a rate code or a spike timing code?”. The coding perspective may help distinguish between rate-based and spike-based theories when it can be shown that one observable carries an insufficient amount of information to account for behavior (Jacobs et al., 2009; Goodman et al., 2013), but this situation is rare. It can also address related questions, such as the timescale of behaviorally relevant information in neural responses (Zuo et al., 2015). But in general, it cannot fundamentally distinguish between rate-based and spike-based theories of the brain, because it focuses on the relation between sensory inputs and neural activity for an external observer and therefore it misses a crucial element of both theories: the way neurons interact with each other. Rates are defined in both rate-based and spike-based theories, but they have different roles: causal in the former, correlational in the latter. Neural variability and low correlations are also typical features of both types of theories, whether they reflect noise or other factors.

The relevant question is thus: *are firing rates atoms of computation or are they just correlates of computation?* Because rates are defined as averages over spikes, this question boils down to whether it is possible to reduce the spiking interactions of neurons to the interaction of rates, in a similar way as the mechanical interactions of particles can be reduced to thermodynamical laws in some cases.

However, there is an important epistemological difference with the case of thermodynamics. Statistical mechanics was developed in an attempt to make two sets of laws consistent: macroscopic laws of thermodynamics and microscopic laws of mechanics. Both were already established, and the question was whether it was possible that one implies the other. The situation is quite different here: laws of spike-based interactions have to some extent been established, but it is an open question whether there are macroscopic laws at all. What has been established to date is that the reduction of spike-based models to rate-based models is not possible in general, and there is no strong indication that it should be in the case of biological neural networks.

If there is neither empirical evidence nor theoretical support for the rate-based view, then why does it have such a broad support in neuroscience? The simple answer is that it is a methodological postulate, before being an empirical hypothesis. A large part of neuroscience theory fits the computationalist framework, in which cognitive functions are described as sequences of mathematical operations defined at a relatively abstract level that is not directly physiological—for example as a combination of linear and nonlinear operations on images (Carandini et al., 1997). Marr (1982) famously argued that we should first try to understand the computational and algorithmic level of cognitive functions, and then independently care about the physical level (neurons), seen as an implementation. It would thus be most convenient if those familiar calculations defined at the algorithmic level could be interpreted as operating on rates, using standard algebra defined on continuous values, and then rate-based calculus could be *implemented* with spikes. It would be convenient, but there is no *a priori* empirical reason why it should be so. There is also no *a priori* functional reason: why would there be any evolutionary pressure for making things simpler for us scientists to understand? In this sense, the rate-based view is primarily a *methodological* postulate.

I have limited this discussion to spiking interactions, neglecting the many other types of interactions, for example ephaptic interactions (Anastassiou et al., 2011), gap junctions (Dere and Zlomuzica, 2012) and graded synaptic transmission (Debanne et al., 2013). This was not to dismiss the potential importance of those interactions, but to specifically analyze the articulation between spike-based and rate-based views. If spike-based interactions cannot be reduced to rate-based interactions, then a fortiori more complex interactions will bring additional difficulties for such a reduction.

How can we make further progress on this question? As the rate-based view is a methodological postulate, and to date mostly an article of faith, the burden of proof should be on the supporters of that view. The strategy is first to show under what conditions it is possible to reduce spike-based models to rate-based models, which is essentially a theoretical task, and then to determine to what extent those conditions are met in the brain. For the defenders of the spike-based view, the strategy should be different. Contrary to what Popper’s logical analysis suggests (Popper, 1959), historical analysis shows that theories are rarely overthrown by empirical refutation alone (Kuhn, 1962), because such refutations may simply lead to refined versions of the theory, sometimes with good reason. New theories tend to replace old theories because they provide a more productive alternative (Lakatos et al., 1978). Rate-based theories are well alive because they fill a methodological need. Thus my suggestion would rather be for defenders of the spike-based view to provide a constructive opposition by developing theories of spike-based computation or dynamics that could favorably replace rate-based calculus, in addition to being empirically sound.

## Conflict of Interest Statement

The author declares that the research was conducted in the absence of any commercial or financial relationships that could be construed as a potential conflict of interest.
